# A retrospective cohort study on lifestyle habits of cardiovascular patients: how informative are medical records?

**DOI:** 10.1186/1472-6963-9-59

**Published:** 2009-04-01

**Authors:** Annemarie J Fouwels, Sebastiaan JH Bredie, Hub Wollersheim, Gerard M Schippers

**Affiliations:** 1 Amsterdam Institute for Addiction Research, AMC, Meibergdreef 5, 1105 AZ, Amsterdam, the Netherlands; 2 Department of Internal Medicine, UMC St. Radboud, Geert Groteplein-Zuid 10, 6525 GA, Nijmegen, the Netherlands; 3 Department of Quality of Care Research, UMC St. Radboud, Geert Groteplein-Zuid 10, 6525 GA, Nijmegen, the Netherlands

## Abstract

**Background:**

To evaluate the vigilance of medical specialists as to the lifestyle of their cardiovascular outpatients by comparing lifestyle screening as registered in medical records versus a lifestyle questionnaire (LSQ), a study was carried out at the cardiovascular outpatient clinic of the university hospital of Nijmegen, The Netherlands, between June 2004 and June 2005.

**Methods:**

For 209 patients information from medical records on lifestyle habits, physician feedback, and interventions in the past year was compared to data gathered in the last month by a self-report LSQ.

**Results:**

Doctors register smoking habits most consistently (90.4%), followed by alcohol use (81.8%), physical activity (50.2%), and eating habits (27.3%). Compared to the LSQ, smoking, unhealthy alcohol use, physical activity, and unhealthy eating habits are underreported in medical records by 31, 83, 54 and 97%, respectively. Feedback, advice or referral was documented in 8% for smoking, 3% for alcohol use, 12% for physical activity, and 26% for eating habits.

**Conclusion:**

Lifestyle is insufficiently registered or recognized by doctors providing routine care in a cardiovascular outpatient setting. Of the unhealthy lifestyle habits that are registered, few are accompanied by notes on advice or intervention. A lifestyle questionnaire facilitates screening and interventions in target patients and should therefore be incorporated in the cardiovascular setting as a routine patient intake procedure.

## Background

Cardiovascular and other non communicable diseases (NCD) account for most of the burden of ill health in Europe [1]. Most of these diseases are associated with common risk factors related to lifestyle habits like smoking, excessive alcohol use, unhealthy eating, and a insufficient physical activity that enhance the risk of developing cardiovascular diseases [2,3]. The WHO regional committee for Europe developed a comprehensive strategy for NCD that includes the active targeting of individuals at high risk and promotes disease prevention programmes[4]. In the Dutch adult general population about, 28% are smokers, 10% consume alcoholic beverages beyond health limits, only one third to one fifth eat fruit and vegetables as recommended, and 46% are overweight. [5]. Several studies in patients with elevated risk or manifest cardiovascular disease indicate favourable effects on cardiovascular risk profiles after a lifestyle change to increase physical activity and reduce body weight [6]. Ornish et al. (1998) demonstrated that, compared to control group, after five years, coronary atherosclerosis was diminished in patients who adhered to intensive lifestyle changes, and 50% fewer cardiac events occurred. [7] In a review of the impact of smoking cessation and smoking interventions in patients with coronary heart disease (CHD), Van Berkel et al. (1999) found that myocardial (re-) infarction declines by 36% in patients who stop smoking after an infarction [8].

The US national high blood pressure education program for the prevention and treatment of high blood pressure recommends weight loss, reduced sodium intake, increased physical activity and limited alcohol consumption [9]. In addition, a diet rich in fruit, vegetables, and low saturated and total fat has also proved to lower blood pressure and reduce cardiovascular disease risk [10] The European societies of hypertension, and cardiology defined observed adverse lifestyle trends among European CHD patients as a cause for concern and urged medical practitioners to place the highest priority on preventive activities that address lifestyle, risk factors, and therapy. The aim is to reduce risk of further coronary and other atherosclerotic events, improve quality of life, and increase chances of survival [11].

In a meta-analysis of methods to modify specific types of behaviour in patients without diagnosed disease, larger effects were produced when information on nutrition, weight, smoking, and alcohol consumption included personal communication [12]. Most patients appreciate their physician paying attention to their lifestyle habits, and most doctors report they are willing to discuss lifestyle habits with their patients [13]. Moreover, physicians are able to promote their patients' healthy behaviour [14].

Nevertheless, the proportion of patients with elevated cardiovascular risk who receive lifestyle attention from their doctors is lower than is generally recommended [15-18]. Although smoking is by far the habit best registered by physicians, it was addressed by general practitioners (GP) in only one-quarter to three-quarters of patient records, according to various studies [19-21,17-19]. In a study by Palonen [22], a patient survey closely resembled the rating of internal medicine residents, they registered smoking status in just over half of their patients and gave advice on smoking cessation to only two-thirds of their smokers. More results were found in Eccles study [23] of lifestyle habits in hypertensive older patients, which shows a gap of 20–50% between habits reported and registered in medical records, even among GP's who say they always inquire about lifestyle habits.

The effect of registration on counselling to stop smoking, was studied by Rothemich [24]. When smoking status was documented as a routine vital sign, brief advice for smoking cessation rose from 53.4% tot 61.9%. Since assessment and monitoring are the first necessary steps before interventions, the registration of risky lifestyle should be mandatory in general health practice, the focus of most studies. We have focused on patients in a cardiovascular setting, where record keeping and attention to risky behaviours are perhaps even more indispensable. This study aims to explore the gap between the documentation, in routine medical care, of lifestyle habits in the medical records and the reporting by patients of their own smoking, alcohol use, physical activity and eating habits. What is the prevalence of unhealthy lifestyle habits as reported by patients? Did physicians, according to their record notes, offer preventive care to their patients with unhealthy lifestyle?

## Methods

The study was conducted from June 2004 until June 2005 in an outpatient clinic for general internal medicine of the University Medical Centre St. Radboud in Nijmegen, a university hospital that is located in the southern part of the Netherlands and covers primarily an urban region. The hospital has both a regional and supra regional function. For the purpose of this study, only the ward for (cardio)vascular illnesses was involved to include patients. The LSQ was completed by participants of a larger study (Fouwels et al., submitted), testing the effectiveness of lifestyle feedback on behavior change. In June 2005, 375 patients had completed the questionnaire and seven patients had refused to do so. The majority of patients, 94%, was born in the Netherlands, one third (33%) followed higher education, and 46% was employed. Out of these patients, 223 had at least one unhealthy lifestyle, their records were searched and 209 were found. We studied medical records on 118 male and 91 female patients, mean age of 55.6 years. All patients were known with manifest cardiovascular disease or with one or more cardiovascular risk factors, who consented to complete the LSQ. A formal informed consent was not required because the central committee on research involving human subjects decided not to judge our study, since the LSQ is only a more structured and detailed way of gathering routine information. The questionnaire was filled out either at the clinic or at home without consuming actual consultation time. About two-thirds of the patients in our study were able to fill out the questionnaire electronically. Eligible patients were those able to understand Dutch, older than 18 years and below 80, and for whom, according to their doctor, change of lifestyle was not overruled by other medical priorities.

The average number of diagnoses per patient was 1.4 *(290 diagnoses/209 patients) *The most frequent diagnoses were: ischemic heart diseases in 18 patients and other heart diseases in 6 patients. CVA/TIA was found in 6 patients and peripheral vascular disease in 41 patients. Diabetes was found in 18; hyperlipidaemia and/or hyperlipoproteinaemia were found in 113 patients, and 60 patients were hypertensive. Adipositas was mentioned as a diagnoses in 7 patients and smoking as a risk factor was mentioned in 4.

### Medical records

To extract data from the 209 medical records, a form was developed to collect lifestyle habits over the past year. Any notes collected about a specific lifestyle habit at the initial visit and at the last year's consultations were collected. We divided the notes into "registrations" of lifestyle habits and "interventions" addressing lifestyle habits. "Registration" was defined as any reference in medical records to smoking, alcohol consumption, physical activity, eating habits, and body weight. "Intervention" was defined as any information, feedback, advice, or referral given in order to change lifestyle by any doctor participating in the routine care of the 209 patients. We used any documented doctors' judgement to define (un)healthy lifestyle, irrespective the presence if quantitative information. Only, if no judgment was available we interpreted any notes on smoking, alcohol use, and physical activity as unhealthy by using cut-off points (see Table 1).

**Table 1:** Cut-off points for unhealthy lifestyle habits applied in medical records and LSQ.

Lifestyle habit	Cut-off for unhealthy in medical records	Cut-off for unhealthy in LSQ
Smoking (package-years, cigarettes per day)	Current smoking	idem
Alcohol use (alcohol; average daily alcohol use in units)	> 2 U/day for females > 3 U/day for males	idem
Physical activity (type and frequency of physical activity)	Once a week or less*	Activity index: < 42 points
Eating habits (intake of vegetables, fruit, fibre and fat)	Sufficient intake*	Fruits/vegetables < 400 g/day fibre ≥ 3 g/MJ; total Fat > 35% of energy intake
Body Weight (body weight in kilograms; Body Mass Index (BMI))	Overweight/underweight* or BMI > 25 kg/m^2^ BMI < 20 kg/m^2^	BMI > 25 kg/m^2^ BMI < 20 kg/m^2^

U = units, a unit equals one standardized glass; g/MJ = gram/megajoule; g = grams; kg/m^2 ^= kilograms/square meters; *judgment by doctor

These included any daily cigarette or cigar use; an average alcohol intake above three units a day for men and above two units a day for women; physical activity with a duration of less than thirty minutes per day and/or less than twice a week; and a Body Mass Index (BMI) above 25 kg/m2. A unit of alcohol is the approximate amount that is routinely served, i.e., 6 oz wine, 12 oz beer, and one fluid ounce of hard liquor.

### Lifestyle Questionnaire (LSQ)

A computerized version of the questionnaire was used (database programme FileMaker Pro 5.5) but a printed version was also available. It took about five minutes to transfer the answers from the printed version to our computer program. Two feedback forms per questionnaire were generated, giving a score visualized in emoticons. One was more detailed, for the physician; one was more general, for the patient. Our 85-item LSQ asks patients about habits of the previous month. It is a compilation of existing questionnaires, and their original cut-offs have been applied. Cut offs used for all 4 studied lifestyle habits are well known and generally accepted by Dutch physicians and patients *alike*. Although the LSQ has not been validated as a compound list, all separate lists have acceptable validity and reliability and have been used to assess smoking, alcohol use, physical activity and eating habits in general health care settings. The LSQ consists of the following sections:

1) Height in meters and weight in kilograms to calculate the BMI.

2) Twelve questions about smoking, of which six concern pack-years, the mean daily number of cigarettes, and the kind of tobaccos smoked; six items of the Fagerström Test for Nicotine Dependence (FTND) [25] were added to measure the effects of situational variables on smoking. A Fagerström-score can be derived using these items to indicate the degree of nicotine dependency of the patient. In this study we used any daily cigarette use as the cut-off point between healthy and unhealthy behaviour.

3) Seven questions about habitual physical activity, i.e., the short version of the International Physical Activity Questionnaire (IPAQ) [26,27], which measures the minutes of physical activity per week and the average daily time spent sitting (e.g., on a chair). In that questionnaire a minute per week is valued 0.4 points for heavy physical activity, 0.2 points for moderate and 0.15 for low activity.

4) Twenty-six questions about eating habits using the Dutch Food Habit Questionnaire concerning fat, fibre, vegetable, and fruit intake [28,29]. Fourteen questions measure total fat and saturated fat intake as a percentage of total energy intake, e.g., asking about whole versus skimmed dairy products, use of cooking oil for baking and roasting, ingestion of meat products, pastry, and candy bars. Six questions measure fibre intake in gram/kilocalories, e.g., asking about use of wholemeal bread, pastas, and brown rice. Six questions measure vegetables and fruit intake in grams per day, asking about consumption of salad, other vegetables, and pieces of fruit.

5) Ten questions about alcohol drinking behaviour from the Alcohol Use Disorders Identification Test (AUDIT) [30-32]. The AUDIT includes three questions to measure the frequency and quantity of alcohol use (and possible binge drinking) and three questions to address symptoms of alcohol dependency. The last four items address possible psychosocial problems.

In order to compare the outcome of our LSQ with that of the medical records concerning unhealthy lifestyle behaviours, the results were categorized according to the cut-off points displayed in Table 1.

### Study evaluation procedure

For each patient who completed the LSQ (N = 209), we reviewed a corresponding medical record. Any written documentation on lifestyle habits in record notes of all consultations for one year preceding completion of the LSQ was observed. Information from the initial consultation was included, even if it occurred more than one year before, since doctors refer tot these baseline notes at succeeding consultations.

### Reliability of data extraction from medical records

To assess inter-observer agreement out of data extraction from medical records, a simple random sample of 20 cases were reviewed independently by a medical doctor and a researcher, each unaware of the other's results. Intra-observer agreement was determined by twice rating the registration of the 20 selected cases, with a three months interval.

Cohen's kappa coefficient could not be computed for the majority of items because one or both raters did not use all categories. For this reason, we report percentage observed agreement instead of kappa's. The amount of observed inter-observer as well as intra-observer agreement was calculated per item. The observed intra-observer agreement was 100% for all items of the medical record registration, the inter-observer agreement 85%. Based on these results, both observed intra- and inter-observer agreement were considered good.

### Calculations and Statistics

Data extracted from the medical records were designated "healthy" or "unhealthy" for each lifestyle habit, based on our judgment or quantitative and/or qualitative information written by the doctors. The extent to which the medical records agreed with the "healthy" or "unhealthy" data generated by the LSQ was assessed by means of a chi-square test. Significance level was set at 0.05. If information in the medical record about a specific lifestyle behaviour was insufficient to score "healthy" or "unhealthy, it was coded only as "registered".

## Results

Just above half of the 209 patients 56.5% (n = 118) were men. Mean age was 55.6 years (SD = 13,5; range: 19–83). Time elapsed since start of last treatment ranged between 0 and 35 years, with a median of 10 years. The last year's record notes represented upon 1 to 20 visits, with a mean of 4 visits per patient and a median of 6. No documentation at all on any of the lifestyle habits was found in 10 records (4.8%).

Table 2 charts the registration of lifestyle habits in the 209 medical records. The notes on a particular lifestyle have been counted for the initial visit and for the last year's records. These comprise the total records mentioned below, as distinct from records for only the last year, which are sometimes considered separately. Notes on smoking behaviour were found in 189 (90.4%) of the total records, and 16 interventions were registered. Last year's notes on smoking were found for 81 patients, and 9 of 20 current smokers received advice from their doctors. One of the advised patients was referred for further treatment.

**Table 2:** Registration of lifestyle habits in the initial visit and the last year's notes (*n *= 209 medical records); values are presented as *n *(%)

Lifestyle habit	Initial visit	Last year's notes	Initial visit plus last year's notes
Smoking	164 (78)	81 (39)	189 (90)
Alcohol use	151 (72)	47 (22)	171 (82)
Physical activity	58 (28)	49 (23)	105 (50)
Eating habits	47 (22)	13 (6)	57 (27)

*n*, number of cases

Alcohol consumption was mentioned in 171 of the total records (81.8%). In the last year's notes, average alcohol consumption, expressed as units a day/week was registered for 47 patients (22.5%). Consumption above the health limit was noted for 7, of whom 5 were given an intervention. Information on physical activity was mentioned in 105 (50.2%) of the total records and 13 interventions were registered. In last year's notes, an assessment of physical activity was found for 49 patients, and a low level of physical activity was noted for 27, of whom 9 received feedback.

Body weight was registered in 206 (98.6%) of the total records. As for eating habits, 57 medical records contained notes on this lifestyle behaviour, and 15 patients were offered advice. Of the last year's records, 13 contained notes on eating habits. An unhealthy diet was mentioned for 3 patients, of whom one was referred.

Table 3 outlines the results of comparing the number of patients labelled as unhealthy in the last year's records vs. those revealed by the LSQ, which reflects last month's lifestyle behaviours. Subjecting the entire sample to x^2 ^tests indicated that in 3 of the 4 lifestyle categories, the number of patients identified as unhealthy was significantly higher when the LSQ was used. Smoking was identified 29 times in LSQ compared to 20 times in the last year's medical records. Alcohol overuse was identified in 42 patients by using LSQ compared with 7 patients in the last year's records. Low physical activity was mentioned for 59 patients using the LSQ, whereas last year's records notes mentioned this 27 times. Unhealthy eating habits were identified by the LSQ in 147 patients compared with 3 patients in the last year's records. Discordant data between LSQ and last year's record notes were found two times for smoking, once for alcohol consumption, and once for physical activity.

**Table 3:** Unhealthy lifestyle habits according to the last year's records versus LSQ (*n *= 209 patients); values are presented as *n *(%)

Lifestyle habit	Unhealthy in last year's record notes	Unhealthy in LSQ	*P*^*a*^, N = 209	X^2^,° N = 209
Smoking	20 (31)	29	0.20	1.480
Alcohol use	7 (83)	42	< 0.001	26.725
Physical activity	27 (54)	59	< 0.001	14.069
Eating habits	3 (97)	147	< 0.001	212.629

*P *= significance value; ^a ^= significance values based on chi-square tests; ° = degrees of freedom = 1

% = fraction underreported unhealthy habits in records compared to LSQ

## Discussion

Assuming that notes in medical records reflect the important topics discussed during consultations, it is remarkable that a thorough evaluation of lifestyle habits is not consistently included. Smoking and alcohol consumption were fairly well registered, but physical activity was documented in only half of the patients, and eating habits just in one out of four patients. The same pattern was found in last year's medical files in which eating habits were by far the worst registered lifestyle behaviour. In most cases the physician paid attention to lifestyle habits at the initial visit, but too often this attention was not routinely repeated. In addition, when unhealthy lifestyles were noted, feedback etc. were only offered in only one of 2 – 4 cases. The exception to this was alcohol use, for which an intervention was usually offered. However, smoking, physical inactivity, overweight, and undesirable food habits [33,34] as well as alcohol abuse are associated with increased risk of developing cardiovascular diseases. Therefore, physicians should pay more attention to all 4 lifestyle habits of their patients [35-37]. Rethans [38] used simulation patients to study record keeping and concluded that only one-third of guidance and advice activities were noted, although Meyboom [39] found that two-thirds of such activities was registered in medical records of general practitioners.

Our study reveals that in everyday clinical practice, complete and meaningful information about lifestyle habits is incompletely registered by medical doctors. As a consequence, their awareness could be suboptimal in regard to the possible influence of unhealthy lifestyle behaviours on the course of a patient's illness. Although the attitude and skills of physicians are essential to support change in patient's lifestyle behaviours, the use of patient self-reported questionnaires can save time and effort and improves the quality of record keeping [40,41] Altogether, we consider that increased effort is worthwhile, considering the potential health benefits for patients. It is particular important for cardiovascular patients, of whom lifestyle behaviours are now often neglected in the consultation room.

As far as practical issues are concerned, the questionnaire takes around 25 minutes to fill out. It can be done before or after the actual consultation time, and results can be made immediately available for both patient and physician. Nevertheless, several limitations of this study should be mentioned. Firstly, our registration consulting room activities was based on analysis of the medical records, and attention might have been paid to lifestyle w/o being recorded. However, it is to be expected that lifestyle changes would be recorded, if considered important. Secondly, the information registered from medical records often contained subjective statements and lacked detail. Therefore, it cannot be ruled out that we underestimated or, less likely, overestimated the presence of unhealthy lifestyle habits. Thirdly, we limited our observations to the initial visit and record notes of the last year's treatment, but not the interval between. Therefore we might have overlooked attention given to lifestyle habits before last year. Lastly, our use of percentage agreement as the only measure for estimating the inter- and intra observer reliability might have been insufficient, because it does not account for chance agreement [42].

## Conclusion

Medical specialists tend to underreport, in routine medical notes, their patients' unhealthy habits. Since those habits are directly related to cardiovascular disease manifestations, this tendency is a cause for concern. Implementation of a self-report lifestyle questionnaire in the care of high-risk cardiovascular patients is feasible and will improve the screening outcome. Further studies are needed regarding the physician awareness of patients' lifestyle, assessing if and how they practice feedback, advice and referral to address lifestyle behaviours.

## Competing interests

The authors declare that they have no competing interests.

## Authors' contributions

AJF participated in the design of the study and drafted the manuscript. SJHB participated in the design of the study and in its coordination. HW participated in the design of the study and helped to draft the manuscript. GMS conceived of the study and helped to draft the manuscript. All authors read and approved the final manuscript.

## Pre-publication history

The pre-publication history for this paper can be accessed here:


